# Accelerated Hydration Site Localization and Thermodynamic Profiling

**DOI:** 10.1021/acs.jcim.4c02349

**Published:** 2025-02-28

**Authors:** Florian B. Hinz, Matthew R. Masters, Julia T. Nguyen, Amr H. Mahmoud, Markus A. Lill

**Affiliations:** † Department of Pharmaceutical Sciences, University of Basel, Klingelbergstrasse 50, 4056 Basel, Switzerland; ‡ Swiss Institute of Bioinformatics, 4056 Basel, Switzerland

## Abstract

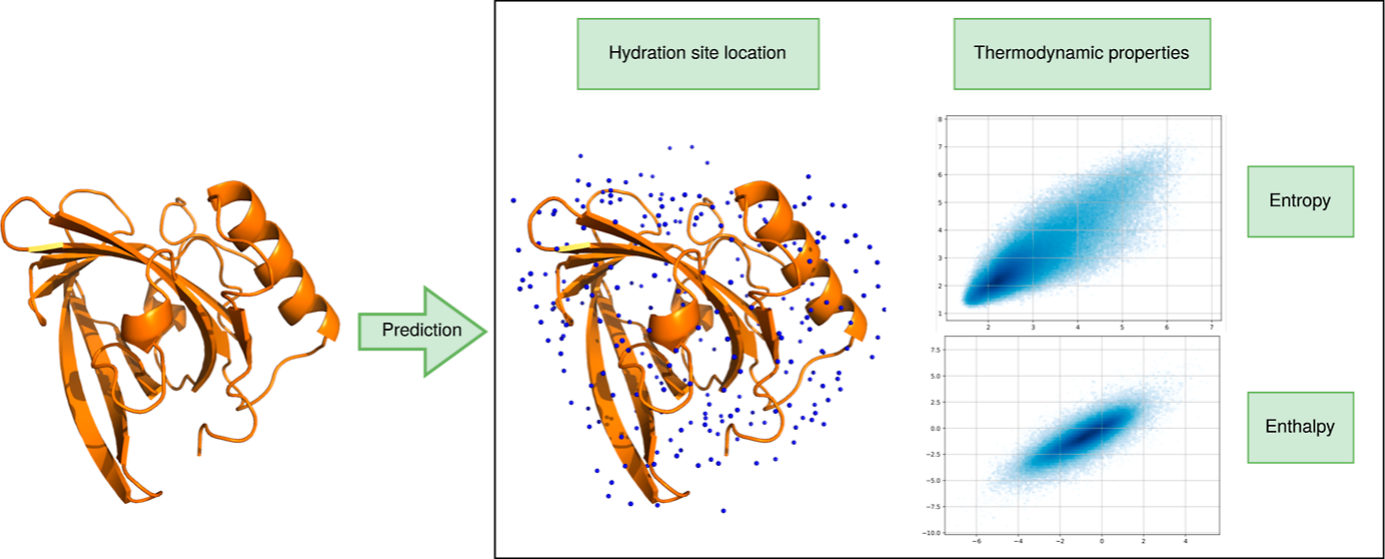

Water plays a fundamental role in the structure and function of proteins and other biomolecules. The thermodynamic profile of water molecules surrounding a protein is critical for ligand recognition and binding. Therefore, identifying the location and thermodynamic properties of relevant water molecules is important for generating and optimizing lead compounds for affinity and selectivity for a given target. Computational methods have been developed to identify these hydration sites (HS), but are largely limited to simplified models that fail to capture multibody interactions or dynamics-based methods that rely on extensive sampling. Here, we present a method for fast and accurate localization and thermodynamic profiling of HS for protein structures. The method is based on a geometric deep neural network trained on a large, novel data set of explicit water molecular dynamics simulations. We confirm the accuracy and robustness of our model on experimental data and demonstrate its utility on several case studies.

## 1 Introduction

Water plays a fundamental role in the structure and function of proteins and other biomolecules. The ubiquitous presence of water in protein environments significantly impacts the stability, dynamics, and interactions with other molecules. In particular, the desolvation of hydrophobic moieties is known to be a major driving force for ligand binding and recognition. Water molecules may also mediate interactions between ligand and protein through the formation of water bridges. Therefore, a complete understanding of the effect of solvation is essential to optimize the binding affinity and selectivity of lead compounds. Beyond small molecules, water also plays an important role in mediating other interactions, such as protein–protein and other biomolecular interactions, which are becoming increasingly important with the development of new biologic products.

Due to its importance and range of potential applications, numerous computational methods have been developed in order to predict the location of hydration sites (HS), localized regions of high water density. However, these methods are usually limited to simplified models that fail to capture the dynamic behavior and multibody interactions inherent in water networks or dynamics-based methods that rely on extensive sampling that is computationally prohibitive for flexible proteins with significant conformational change. Furthermore, simply identifying the position of likely water molecules is insufficient for ligand optimization since the thermodynamic profile of individual water molecules dictates whether displacement by a ligand is energetically favorable or not. Computational methods also exist for estimating the thermodynamic properties of HS but suffer from the same accuracy/cost trade-off between static- and dynamic-based methods.

In this article, we introduce a generative deep learning model that is capable of fast and accurate hydration site identification and thermodynamic profiling. Unlike previous methods, our model is capable of predicting HS in one-shot with near dynamics-level accuracy. The model is able to resolve complex multibody interactions in a fixed-time and propose physically valid water networks along with associated enthalpic and entropic contributions. It is based on an equivariant transformer network and is trained to match the explicit distribution of HS on a protein surface. Training was done using a novel data set of WATsite simulation results from thousands of diverse protein systems, representing a high level of robustness and generalizability across protein space. Our method was validated using a difficult holdout set of unseen protein sequences in addition to high-quality crystallographic waters. We further demonstrate and discuss our model’s utility in a number of interesting drug and protein design scenarios.

## 2 Background and Related Methods

### 2.1 Experimental Determination

Water locations can be resolved using several experimental methods including X-ray/neutron crystallography,^1^ cryo-EM,^2,3^ and NMR.^4^ Crystallography is the most common technique with more than half of the experimental structures containing at least one water molecule.^5^ However, there are a number of technical challenges associated with the determination of water locations, leading to certain limitations. Most of these challenges arise from the complex multibody interactions and high mobility inherent to water molecules. Experimental determination is often limited to the first shell of waters, especially on the solvent-exposed surface and not buried within the protein. Furthermore, experimental approaches have limited resolution, and water positions often have nonideal interaction geometry. Most importantly, the experimental determination of water locations does not quantify thermodynamic properties such as enthalpy and entropy.

### 2.2 Computational Methods

While experimental determination of water structure is indispensable for providing the ground truth, they are limited by real-world constraints such as time, cost, and technical feasibility. To overcome these challenges, a number of computational methods have been developed to predict the location of water molecules around an existing protein structure. These methods can be largely grouped into two categories: static and dynamic. Static methods assume that the water network has a constant position and neglect any slowly resolving water–water or water–protein interactions as well as any protein movement or flexibility. Among the static methods, most fall into the category of knowledge-based, such as 3D-RISM,^6−8^ AQUARIUS,^9^ SZMAP,^10^ and WaterFLAP,^8,11,12^ geometry-based, such as AcquaAlta,^13^ Auto-SOL,^14^ HADDOCK,^15^ WarPP,^16^ and WATGEN,^17^ or energy-based, such as Fold-X^18^ or WaterDock.^19^

Dynamic methods often employ molecular dynamics (MD) simulations, which utilize an explicit water model and protein force field. By using MD simulations, one can generate a trajectory detailing the atomic behavior of water with other molecules in solution. This trajectory can be later analyzed to identify regions of high water density, characterize the thermodynamics of these sites, and understand detailed interactions involving water. However, the limitation of dynamic methods is that they are computationally and time intensive, especially due to the calculation of large number of nonbonded interactions that is required by the explicit water simulation. Furthermore, in order to ensure convergence of the water density within a reasonable time, strong positional constraints are added to the protein solute, removing any flexibility and perturbation to the initial protein structure.^20,21^ While these constrained results can still be useful in guiding structure-based drug design for particular targets, we know that protein flexibility plays a significant role in most drug targets and that even small perturbations to protein structure can lead to drastically different hydration shell structure.^22^ Therefore, dynamic methods are still largely limited to single protein structures and require either multiple runs or time-consuming, extensive runs in order to achieve proper convergence. Among the dynamic methods are HydraMap,^23^ HyPred,^24^ MobyWat,^25,26^ WaterMap,^8,27^ and WATsite.^28−30^

More recently, some works have employed machine learning and deep neural networks toward the prediction of hydration shell structure. For example, several groups have used convolutional neural networks that work on 3D grids in order to predict the water density such as GalaxyWater-CNN,^31^ HydraProt,^32^ and Ghanbarpour et al., 2020.^33^ In another work, Park developed an SE(3)-equivariant network that is capable of directly predicting positions of water molecules given predefined probe locations.^34^ Lastly, SuperWater is a diffusion-based model designed to generate crystal waters given the protein structure.^35^ While these methods take a step toward the development of a one-shot hydration site predictor, they still fall short either by training on sparse and poorly modeled crystallographic data or by not considering the thermodynamic contributions of HS.

## 3 Methods

The prediction of HS with associated thermodynamic properties is based on two separate models. The first model predicts the hydration site location and is explained in section 3.1. The predicted coordinates then serve as input to the second model, which estimates the corresponding changes in entropy and enthalpy when a water molecule transitions from bulk solvent to the hydration site (see section 3.2). The code and a reference to the training data are provided at https://github.com/lillgroup/HydrationSitePrediction.

### 3.1 Location Prediction

**Figure 1:**
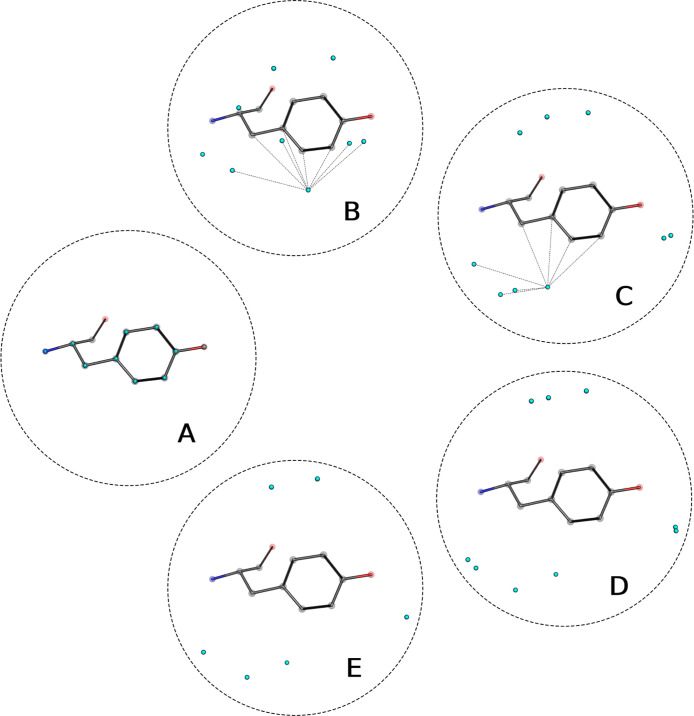
Schematics displaying the coordinates prediction of HS. (A**)** Initially, we position a water prediction (light blue spheres) at every protein heavy atom exceeding a certain cutoff of solvent exposure (SASA greater than 0.1). An equivariant attention layer is applied to the distance-based graph topology to perturb the water predictions. (B,C**)** The distance-based graph topology is updated by now also considering water predictions as graph nodes. An equivariant attention layer perturbs the water predictions. (D) After the application of five layers, predictions of low certainty are filtered out and a clustering algorithm is applied to end up at the final water predictions (E).

The procedure of learning the hydration site locations is inspired by our previous work^36^ and schematically shown in Figure 1. The input data consist of the protein atom coordinates (excluding hydrogen atoms) and the associated feature vectors. The feature vector is 92 dimensional consisting of a one-hot encoding of the atom type (38 dimensions), a one-hot encoding of the residue type (21 dimensions), a radial basis function expansion of the solvent accessible surface area (SASA) value (32 dimensions), and a one-hot encoding for the node type (atom or predicted water, 1 dimension). The coordinates of the protein’s heavy atoms with solvent exposure serve as the initial placement for water predictions. More specifically, the oxygen atom of an initial water molecule is placed at the same location as a protein atom (excluding hydrogen atoms) if the SASA value of the protein atom exceeds 0.1 (see Figure 1A). We observed that increasing the SASA cutoff for initial placement led to reduced model performance, whereas lowering the cutoff increased the computational cost without improving the predictive capacity.

A graph structure is constructed from the positions of the heavy atoms by connecting any two nodes (i.e., atoms) via an edge, if their distance is less than 6 Å. The distance cutoff value of 6 Å for the edge construction was chosen based on a hyperparameter optimization on the training and validation set. The edge feature encoding consists of a radial basis function expansion of the edge length (32 dimensions). We apply a first equivariant attention layer to the graph structure to obtain updated locations of the water molecule nodes. The protein atom positions are left unchanged. The graph topology is updated by also including the nodes representing the water molecules and again constructing edges between nodes that are less than 6 Å apart. Five equivariant attention layers are applied to perturb the nodes representing water molecules, with the distance-based graph topology being updated before applying each layer (see B,C in Figure 1). The network is trained by minimizing a loss function that resembles the Kullback–Leibler divergence between two Gaussian mixture models: once the mixture components are defined via the ground truth water coordinates and once via the predicted coordinates. Details of the loss function are provided in the following paragraph.

#### 3.1.1 Loss Function

The equivariant graph neural network as discussed in section 3.1 outputs tuples  for *j* ∈ {1,···,*n*} and  the number of initially predicted water nodes. The first element  represents the coordinates of the predicted hydration site (see D in Figure 1), while *w*_*j*_ ∈ [0, 1] corresponds to an associated certainty weight of the prediction. Defining for *j* ∈ {1,···,*n*} the normalized certainty weights as
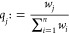
1we consider the tuples  as a representation of components of a Gaussian mixture distribution as follows
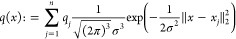
2where the standard deviation is chosen as σ = 0.5 Å. For a given protein in the training set, we refer to the simulated HS based on WATsite as “reference HS”. Let  be the  reference HS with associated occupancies of at least 0.5. Similarly, as in 1 and 2, we define
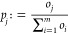
3and consider the reference HS representing a Gaussian mixture distribution as follows
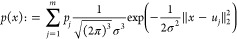
4

As a simplified surrogate for the symmetrized Kullback–Leibler divergence KL(*p*|*q*) + KL(*q*|*p*), we choose
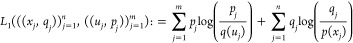
5

We found it beneficial to introduce a further loss term, penalizing the concentration of weights
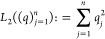
6

The total loss function consists of a weighted sum of *L*_1_ and *L*_2_
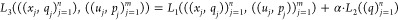
7for some hyperparameter α > 0.

#### 3.1.2 Clustering

At inference time, we first filter out predictions with certainty weights lower than a cutoff of *w*_c_ = 0.035 to allow the identification of well-separated clusters. To the remaining predictions, we apply an agglomerative clustering method as implemented in,^37^ using the default linkage method (“ward”) with a cluster linkage distance threshold of 2 Å. The agglomerative clustering algorithm will merge two clusters if their distance (in the sense of the “ward” function) is less than the cluster linkage distance threshold. The hyperparameters *w*_c_ and the cluster linkage distance were chosen based on visual inspection and evaluation on the validation set.

Per cluster, we calculate its weighted mean and the associated certainty weights as follows: Let  and
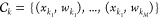
8be the *k*’th cluster determined by the clustering algorithm, consisting of the  coordinate and weight predictions indexed by . We define the weighted cluster mean with associated certainty weight as
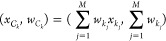
9

We consider  a predicted hydration site location (see 1D) if the associated certainty weight *w*_C_k__ exceeds a threshold of 0.1.

### 3.2 Prediction of Entropy and Enthalpy Changes

**Figure 2:**
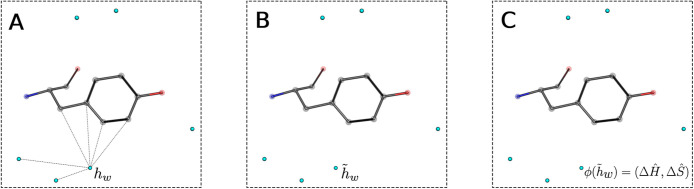
Schematics displaying the prediction of changes in entropy and enthalpy due to the transfer of a water molecule from the bulk solvent to the hydration site. (A) The input consists of coordinates and feature vectors for protein heavy atoms and HS. The edges are constructed based on distances. (B) A graph attention network is applied and outputs a modified feature vector per graph node. (C) A feed forward network is applied to the feature vectors of the water nodes. The output represents the enthalpy and entropy differences for the specific hydration site.

We aim to predict the change in enthalpy Δ*H* and entropy Δ*S* associated with the transition of a water molecule from the bulk solvent to a specific hydration site. Figure 2 schematically shows the method for learning Δ*S* and Δ*H*. First, a graph is constructed with nodes representing heavy atoms of the protein and HS. During training, HS from WATsite with occupancy values greater than 0.3 are considered nodes. The edges are constructed based on a distance cutoff of 8 Å, ignoring intramolecular edges of the protein. This choice of edge construction was based on evaluating the model performance on the training and validation sets for varying distance cutoffs and including or excluding intramolecular edges of the protein. The feature vectors associated with the nodes are similarly constructed as for the location prediction model (see section 3.1). The feature vectors are updated by applying three layers of a graph attention network as introduced in^38^ (see Figure 2 B). A feed forward network is applied to each feature vector to end up at a two-dimensional output that represents the predicted differences in entropy and enthalpy due to the transfer of a water molecule from bulk solvent to the hydration site (see Figure 2C).

#### 3.2.1 Loss Function

Let  denote the number of HS with an occupancy of at least 0.5. We index these HS by *i* ∈ {1, ..., *n*} and denote by Δ*S*_*i*_ and Δ*H*_*i*_ the differences in entropy and enthalpy, respectively, corresponding to the change in entropy and enthalpy associated with the transitioning of a water molecule from the bulk solvent to hydration site *i*. The details about the calculations of Δ*S*_*i*_ and Δ*H*_*i*_ from molecular simulations are discussed in section 3.3.

The loss function during training consists of the sum of the mean squared errors of entropy and enthalpy over all HS with occupancy of at least 0.5
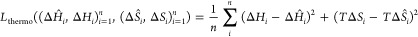
10where  is the number of HS with occupancy of at least 0.5 and *T* = 300 K is the temperature. By  we denote the model prediction for the change of enthalpy Δ*H*_*i*_ of the hydration site *i* ∈ {1,···,*n*} due to the interaction with protein. Similarly, the prediction of the target value entropy Δ*S*_*i*_ is denoted by  for *i* ∈{1,···,*n*}.

### 3.3 Data Set

The training data was prepared using WATsite^28,39^ and the number of proteins contained in the data set was 4148, with occupancy and thermodynamic values for the HS. For the selection of the data set, 19,443 proteins from PDBbind^40,41^ were grouped by their UniProt ID and one representative structure was randomly picked from the group to prevent redundancy. The test set was selected to be as different from the validation and training set as possible, to evaluate the model’s ability to generalize new targets. This was done with the sequence-similarity-based splitting strategy, in which the pairwise sequence similarity of all proteins was computed by MMSeqs2^42^ and all PDBs up to a certain similarity threshold were grouped together. The similarity cutoff in this case was 35% and splitting was done until a 75:25 train test ratio was achieved. Ultimately, the train set contained 2766 proteins; the test set contained 1083 proteins, and the validation set contained 305 proteins.

For the MD simulation the cocrystallized small molecules, waters and ions were removed with Schrödinger’s Protein Preparation Wizard.^43^ Simulations were performed with Schrödinger’s Desmond according to its standard NPT relaxation protocol^44^ using the OPLS4 force field.^45^ The production simulations ran at 300 K for 20 ns each, with a 50 kcal mol^–1^ Å^–2^ large positional restraint applied to all heavy atoms. Every 20 ps, snapshots were taken, resulting in 1000 frames per simulation for analysis. Using VMD,^46^ the MD simulations were aligned and recentered and the HS calculated from each simulation trajectory using the WATsite program. The water molecules were tracked throughout the simulation, and their density is discretized onto a grid with resolution of 0.25 Å. A hierarchical clustering algorithm clustered the grid into HS with a radius of 1 Å. Water molecules belonging to each hydration site are extracted from the simulation, allowing for the calculation of thermodynamic properties. The change in enthalpy (Δ*H*) was determined based on the average of van der Waals and electrostatic interactions between each water molecule inside a hydration site with the protein and other water molecules compared to the value in the bulk solvent.
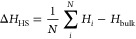
11

Meanwhile, the entropy change was estimated by integration of the external mode probability density function (PDF, *p*_ext_(*q*)) of the water molecules’ translational and rotational motions during the MD simulation via the following formula
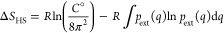
12With *R* being the gas constant, *C*° is the concentration of pure water (1 molecule/29.9 Å^3^). From entropy and enthalpy differences, the Gibbs free energy of the desolvation of HS could be calculated using the following formula: Δ*G* = Δ*H* – *T*Δ*S*. It represents the temperature (*T*) dependent change of entropy and enthalpy of a water molecule inside a hydration site, when it is transferred from the bulk solvent into the hydration site of the protein cavity.^47^

## 4 Results and Discussion

### 4.1 Hydration Site Prediction

In the following, we present the results of the model discussed in section 3.1 on the test set (similar results for the training set are provided in the Supporting Information). We evaluate the prediction performance in terms of the **G**round **T**ruth **R**ecovery **R**ate (**GTRR**) and **P**rediction **H**it **R**ate (**PHR**) as defined in the eqs S1 and S2 of the Supporting Information. Table 1 shows the GTRR and PHR for a cutoff value of 0.5 and 1.0 Å. The model recovers 59% of the reference HS at a cutoff level of 0.5 Å divergence. If we increase the cutoff to 1.0 Å, then 80.2% of HS are recovered. On the other hand, a proportion of 48.3% of predictions is closer than 0.5 Å to a reference hydration site and 65.9% are closer than 1.0 Å. This performance is comparable to other hydration site prediction methods.^5^ However, these comparisons use different test sets and are often limited to a smaller number of systems than we evaluate on here.

**Table 1:** GTRR and PHR on WATsite Test Set

cutoff (Å)	ground truth recovery rate	prediction hit rate
0.5	59.0%	48.3%
1.0	80.2%	65.9%

We consider a hydration site to be in the first layer if it is no further distant than 3.5 Å from a non-hydrogen atom of the protein. Otherwise, the hydration site is in the second layer. Table 2 shows the GTRR for first and second layer HS at cutoff levels of 0.5 and 1.0 Å. We observe that the model very successfully predicts first layer hydration site coordinates with a GTRR of 62.5% and 84.5% for and 0.5 and 1.0 Å, respectively. The second layer HS are substantially more challenging to predict correctly, as only 15.2% and 26.9% of reference HS are recovered at a tolerance cutoff of 0.5 and 1.0 Å, respectively. These results indicate a clear distinction in the model’s predictive accuracy between first and second shell HS. First layer HS are strongly influenced by electrostatic and steric effects, often forming direct hydrogen bonds with the protein, making them more predictable. Second layer HS have reduced accuracy, due to their weaker, indirect interactions with the proteins. This leads to a greater mobility of the water and less localized water density.

**Table 2:** GTRR for First and Second Layer

cutoff (Å)	GTRR first layer	GTRR second layer
0.5	62.5%	15.2%
1.0	84.5%	26.9%

We further investigate the recovery performance for HS differentiated based on occupancy in Table 3. As one would naturally expect, the GTRR increases with higher occupancy, meaning that more stable HS are easier to predict. The GTRR is highest for HS of an occupancy between 0.8 and 0.9 with 70.0% and 91.0% for a divergence cutoff of 0.5 and 1.0 Å, respectively. For the HS of highest occupancy (between 0.9 and 1.0), the model receives almost the same performance with 69.7% and 90.8%, respectively.

**Table 3:** GTRR at Different Occupancy Levels

	occupancy				
	**[0.5,0.6]**	**[0.6,0.7]**	**[0.7,0.8]**	**[0.8,0.9]**	**[0.9,1.0]**
**cutoff (Å)**					
*r* = 0.5	42.1%	57.2%	65.1%	70.0%	69.7%
*r* = 1.0	62.4%	79.4%	86.9%	91.0%	90.8%
*r* = 1.5	70.4%	86.3%	92.5%	95.7%	94.4%
*r* = 2.0	75.1%	89.5%	94.8%	97.3%	95.6%

As shown in Table 4, the results significantly improve for low occupancy HS if we just consider the HS of the first layer.

**Table 4:** First Layer Waters: GTRR at Different Occupancy Levels

	occupancy				
	**[0.5,0.6]**	**[0.6,0.7]**	**[0.7,0.8]**	**[0.8,0.9]**	**[0.9,1.0]**
**cutoff (Å)**					
*r* = 0.5	48.5%	60.6%	67.0%	70.9%	69.9%
*r* = 1.0	71.0%	83.6%	89.0%	91.9%	90.9%
*r* = 1.5	79.4%	90.5%	94.5%	96.5%	94.5%
*r* = 2.0	83.7%	93.3%	96.5%	97.9%	95.7%

### 4.2 Thermodynamic Profiling

In the following, we evaluate our model for thermodynamic profile prediction as introduced in section 3.2. Given the coordinates of the reference HS, we investigate how accurately the model can recover the enthalpy and entropy differences given by the explicit-water MD simulations with WATsite. A case study where we predict thermodynamic properties based on predicted hydration site locations is provided in subsection 4.3.5.

**Figure 3:**
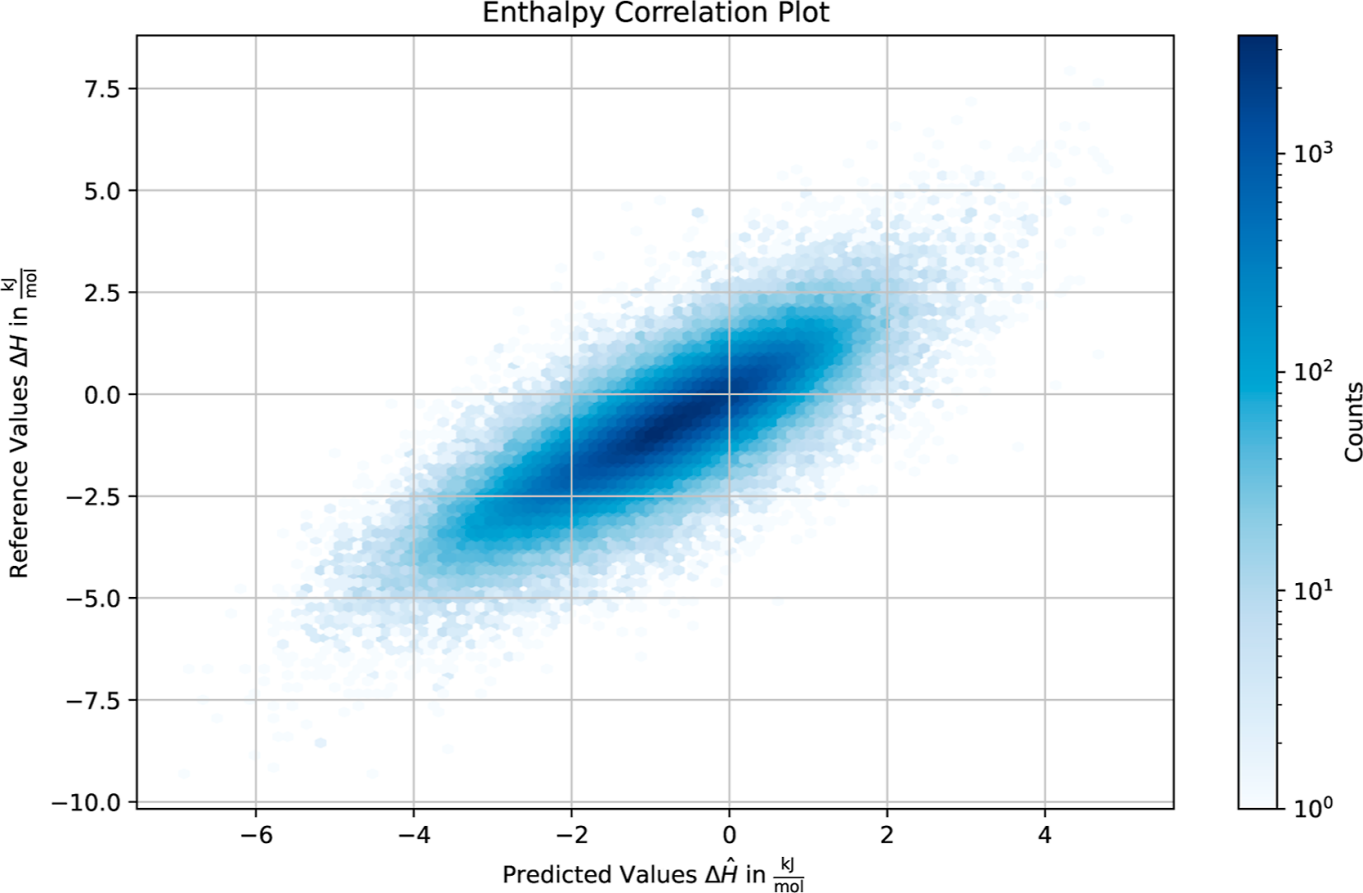
Hexbin plot showing the correlation between the predicted enthalpy differences and the enthalpy differences obtained from WATsite for the test set.

**Figure 4:**
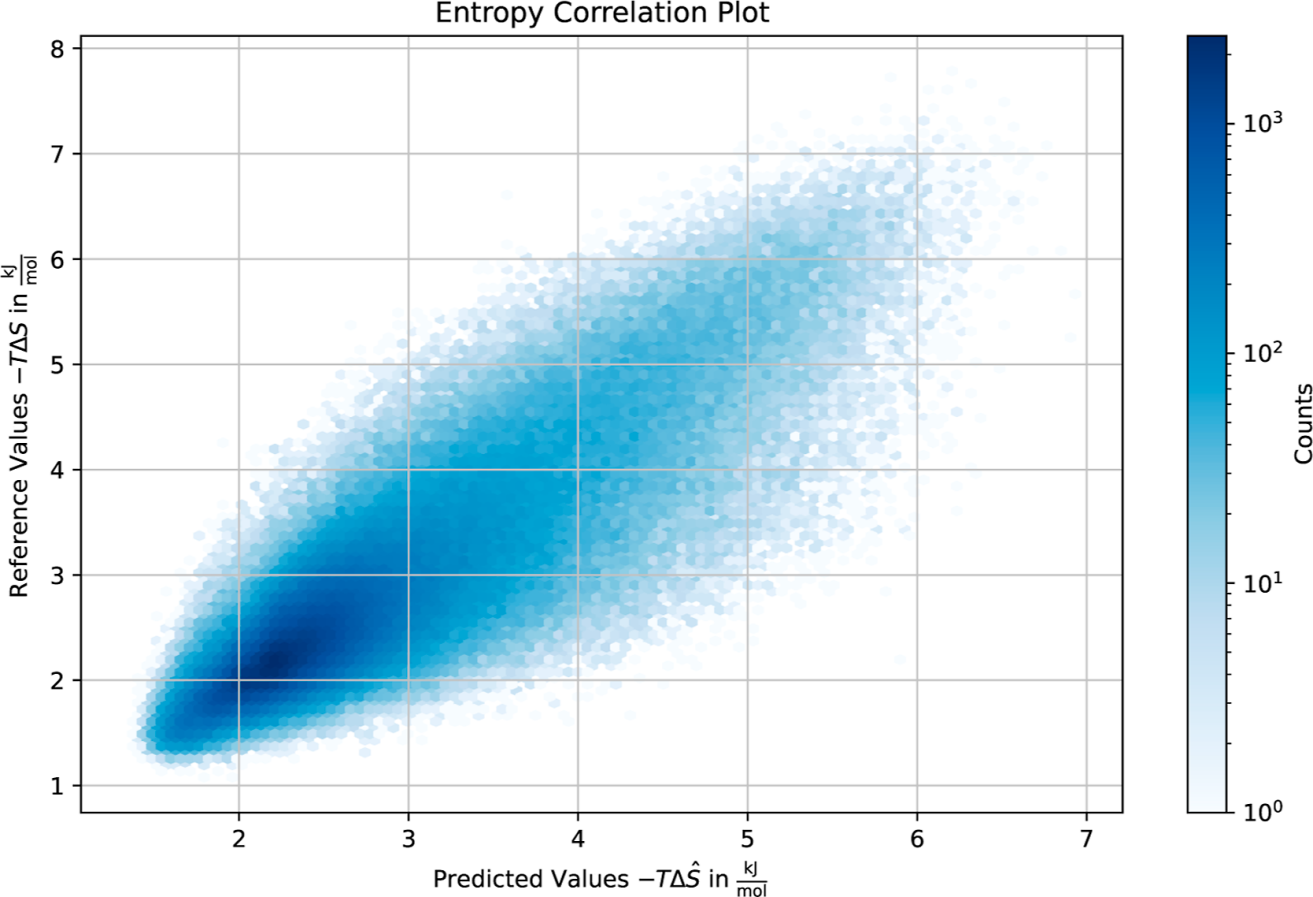
Hexbin plot showing the correlation between the predicted entropy differences and the entropy differences obtained from WATsite at *T* = 300 for the test set.

Table 5 shows the results for both enthalpy difference Δ*H* and entropy difference – *T*Δ*S* in terms of mean squared error and *R*^2^ value on the test set. The correlation between predicted and reference values for the enthalpy difference and entropy difference is visualized in hexbin density plots in Figures 3 and 4, respectively (and Figures S1 and S2 for the training set). We observe that given the reference hydration site coordinates, the correlation between model predictions and the corresponding simulated target values is high, with *R*^2^ = 0.75 for the entropy difference and *R*^2^ = 0.70 for the enthalpy difference. Interestingly, there seems to be an increasing uncertainty for predictions in the case of higher entropy difference values. High-entropy waters are often characterized by greater mobility, weaker structural organization, and fewer hydrogen bonds. This leads to a broader distribution of possible locations and orientations of the water molecule. Consequently, these less ordered HS are harder to predict accurately.

**Table 5:** Coefficient of Determination (*R*^2^) and Mean Squared Error (MSE) between Prediction and Reference Values for Both Enthalpy and Entropy Differences on the Test Set

	**–***T***Δ***S*	**Δ***H*
**MSE** in	0.22	0.38
***R***^2^	0.75	0.70

### 4.3 Case Studies

In order to investigate the accuracy and behavior of the model more intently, we looked at five specific systems: Disulfide Catalyst DsbA, Triosephosphate Isomerase, Heat Shock Protein 90 (HSP90), Clarin-2, and Major Urinary Protein (MUP). The first two focus on conserved water networks that are important for structure and function as well as the effect of point mutations. The HSP90 example focuses on conformational changes ranging from side-chain rotation to loop reorganization. Clarin-2 was used to test the ability of the model to predict hydration information using the AlphaFold3 structures of unresolved membrane-bound proteins. Finally, in the MUP case study, we apply thermodynamic predictions to the case of protein–ligand binding and show that a strong correlation can be established between the estimated free energy of desolvation and experimentally measured affinity values for a series of ligands. None of these systems was present in the training set.

#### 4.3.1 Disulfide Catalyst DsbA

**Figure 5:**
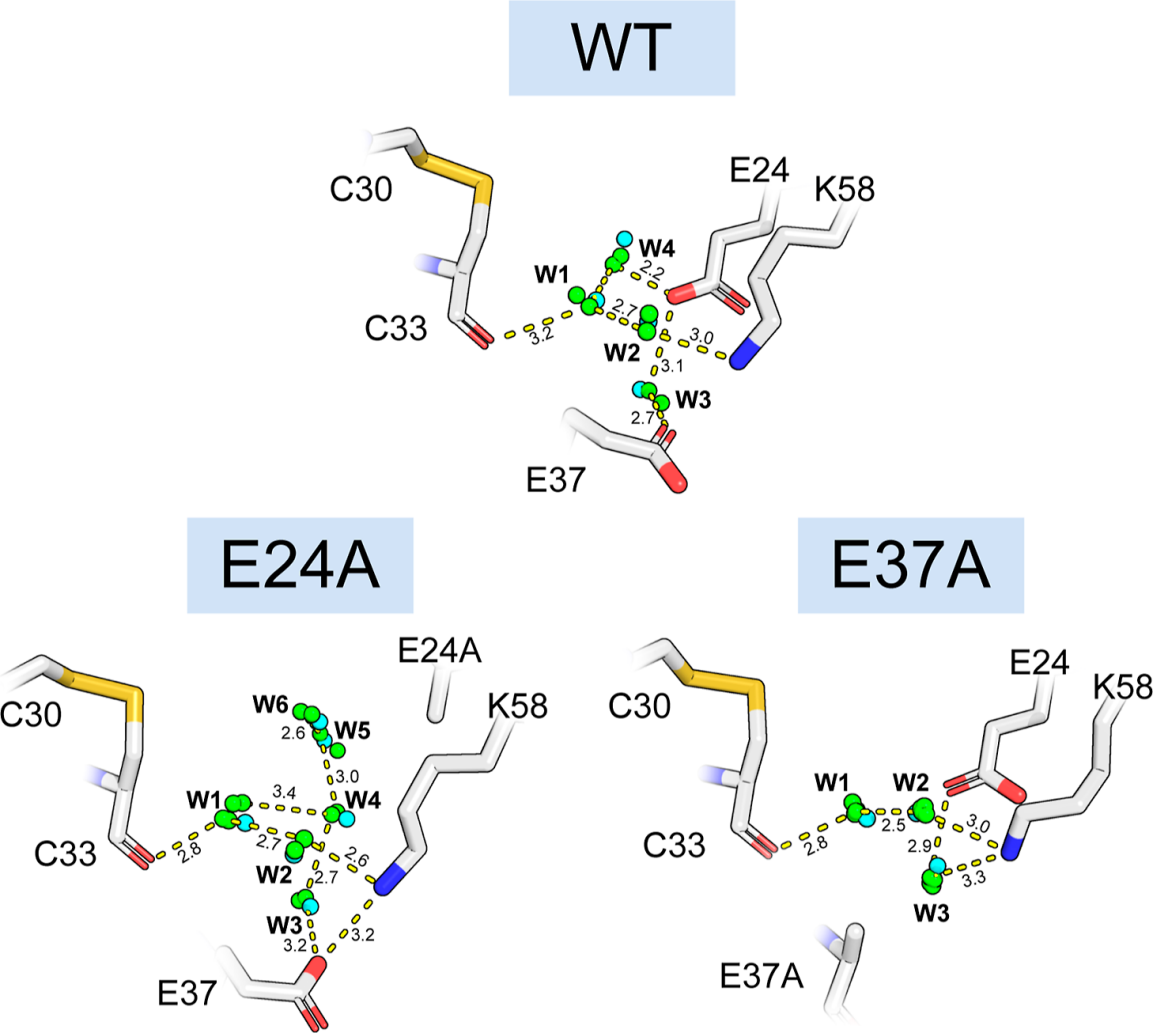
Buried water network important for the activity of disulfide catalyst DsbA. We evaluated the wildtype (PDB: 5QO9) as well as two mutants known to disrupt the conserved water network, E24A (PDB: 8EQR) and E37A (PDB: 8EQQ). Crystal waters are shown from all superimposed chains in green. Hydration site predictions are shown in cyan. Relevant distances are shown and labeled with their length in Angstrom. Predictions beyond the selected crystal HS are not shown.

Disulfide Catalyst DsbA is an important enzyme for the formation of disulfide bonds, which are present in many proteins entering the secretary pathway.^48^ Importantly, disulfide catalysts contain a conserved buried water network within the active site that is essential for its function. It is believed that the water network acts as a “proton wire” that shuffles protons from the bulk solvent to the catalytic cysteine residues. Previous studies have investigated the effects of point mutations within the active site using X-ray crystallography.^49^ In this experiment, we aimed to test whether our model could correctly generate the conserved water network structure and respond accurately to point mutations. The results, shown in Figure 5, show a high level of accuracy for both the wild type and both point mutations (E24A and E37A). In each case, we were able to correctly identify all of the HS within 1.0 Å. These results demonstrate that the model is capable of predicting structurally and functionally important water networks with a high degree of accuracy. The model also appears robust and generalizable by its ability to accurately respond to point mutations. A common fault of deep-learning-based models is their tendency to overfit and inability to behave accurately outside of the training data distribution.^50^ However, the results indicate that our model is not overfit on any particular protein folds and can respond to small changes in protein structure. Lastly, the predicted HS appear to be physically valid with no steric clashes and reasonable interatomic distances.

#### 4.3.2 Triosephosphate Isomerase

**Figure 6:**
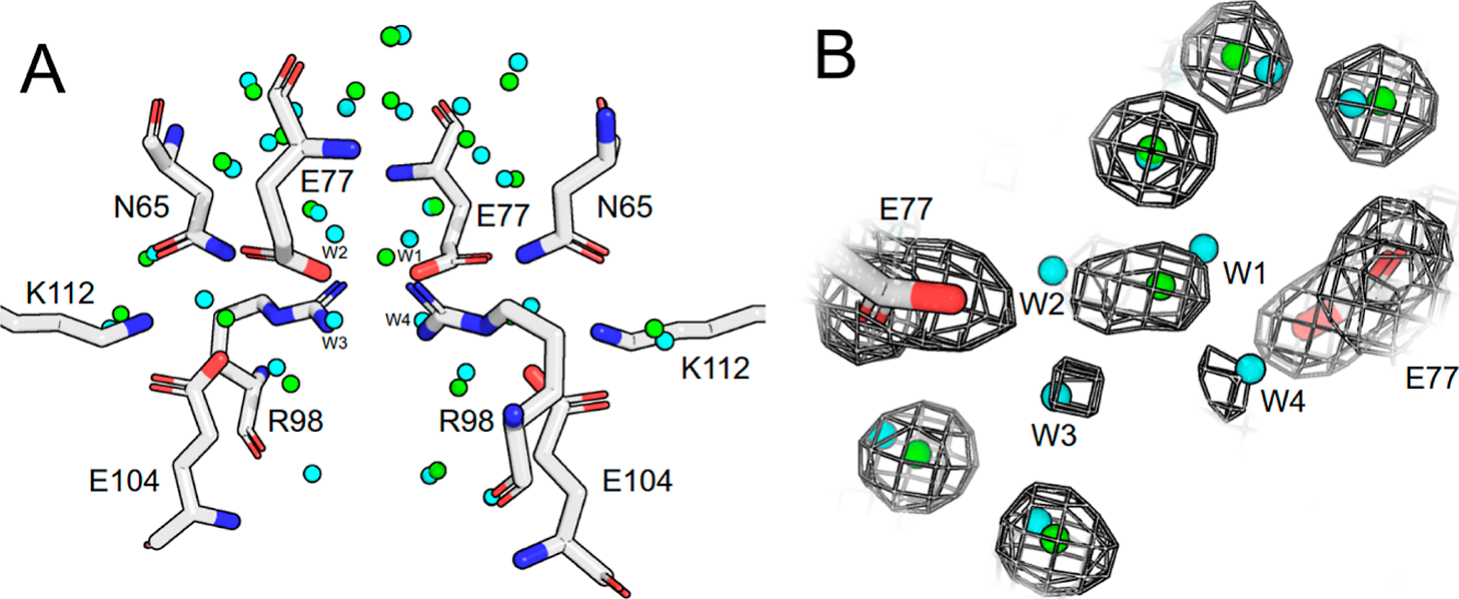
Conserved water network at the dimer interface of triosephosphate isomerase. (A) Residues at interface of subunits A (right) and B (left) with crystal waters (green) and predicted HS (cyan). (B) Focused view of the single failure at center of interface with 2fo-fc map shown as mesh with isovalue of 1.0.

Triosephosphate isomerase is a dimeric enzyme involved in the glycolysis pathway and is found in nearly all organisms.^51^ Like in the previous case study, triosephosphate isomerase also contains a conserved water network. However, in this case, the water network is present at the interface of the homodimer structure and not buried within the protein. The predictions showed very strong agreement with experimental data with 20/21 crystal waters predicted within 1.0 Å (Figure 6A). Upon further inspection of the single failure, our proposed HS occupy unmodeled regions of electron density, potentially offering a better fit than the PDB deposited structure (Figure 6B).

#### 4.3.34.3.3 Heat Shock Protein 90

**Figure 7:**
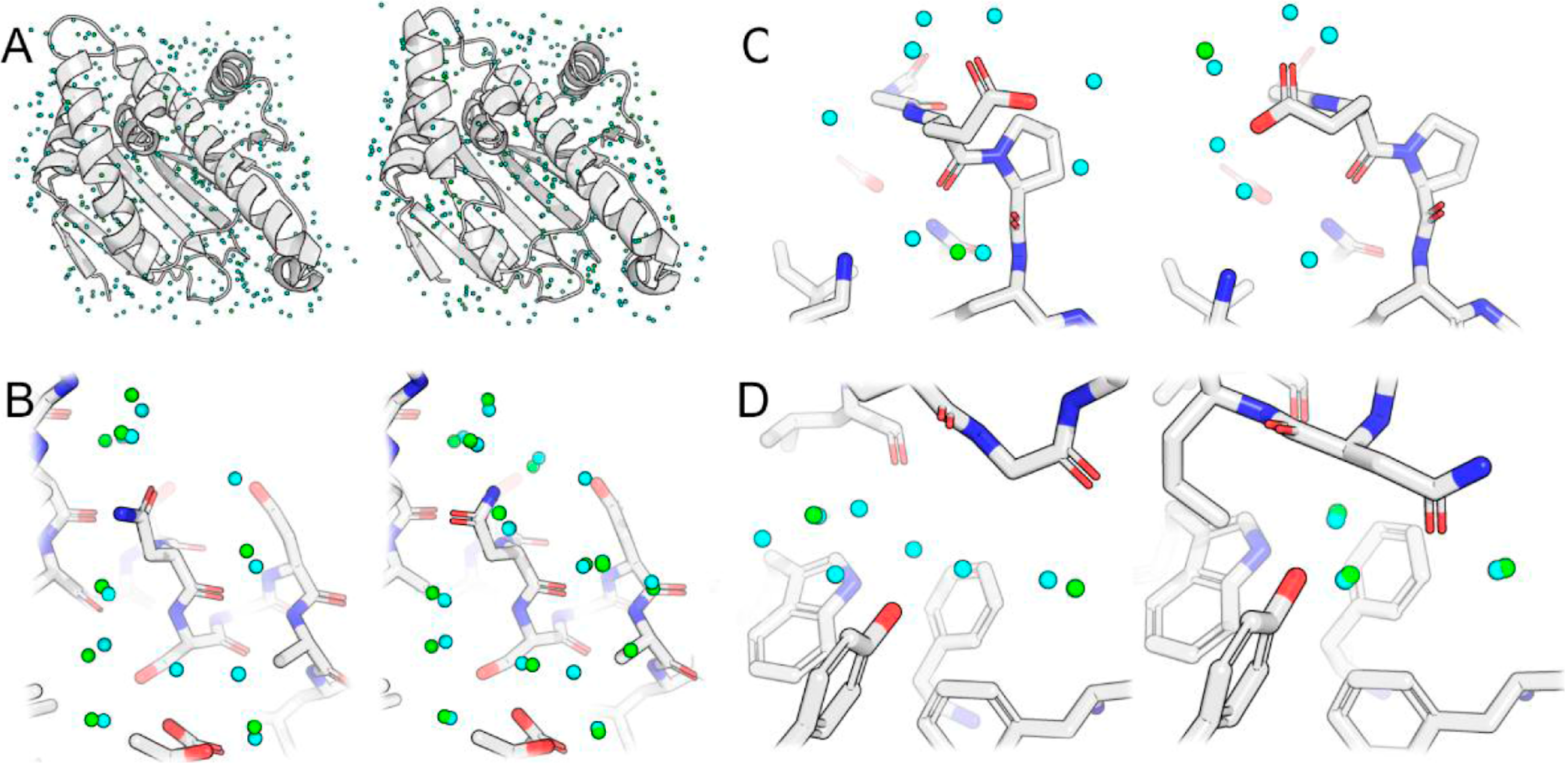
Comparison of HSP90 in two conformations from PDBs 2QFO and 2WI7 on the left and right, respectively. (A) Full protein structures with all crystal waters (green) and predictions (cyan). (B) Focused view on region with similar conformation in both structures and strong agreement with crystal waters. (C) Example of flexible side-chain in different conformations with corresponding predicted waters. (D) Several HS occupying the open pocket of 2QFO are displaced or stabilized by the α-helix3 side-chains seen in the 2WI7 loop-in structure.

The location of HS is highly dependent on protein conformation.^21,22^ Therefore, it is essential that the model is capable of responding to changes in the protein, ranging from small perturbations to large-scale conformational changes. To this end, we predicted HS for HSP90 in two conformations which exhibits both small and large conformational change (Figure 7). In regions where the conformations are similar, the model predicted similar water networks that exhibited strong agreement with crystal water locations (Figure 7B). This confirms that distant changes in protein structure does not result in undue changes to the hydration site prediction. In the case of a flexible side-chain that can adopt multiple possible conformations, the model was able to adapt to the change, maintaining proper hydrogen bond distances and avoiding steric clashes (Figure 7C). This finding demonstrates the robustness of our model in responding to small changes and accurately modeling atomic interactions. This also shows that our model is not overfit to any particular conformation and the network has not simply memorized the placement of HS based on sequence alone. HSP90 also exhibits a larger conformational change which is α-helix3. In 2QFO, the α-helix is intact, while in 2WI7 the helix unfolds in the middle and adopts a “loop-in” conformation. The model accurately responds to the large conformational change and is able to detect both the displacement and the stabilization of several sites, further supported by crystallographic waters (Figure 7D).

#### 4.3.4 Clarin-2

**Figure 8:**
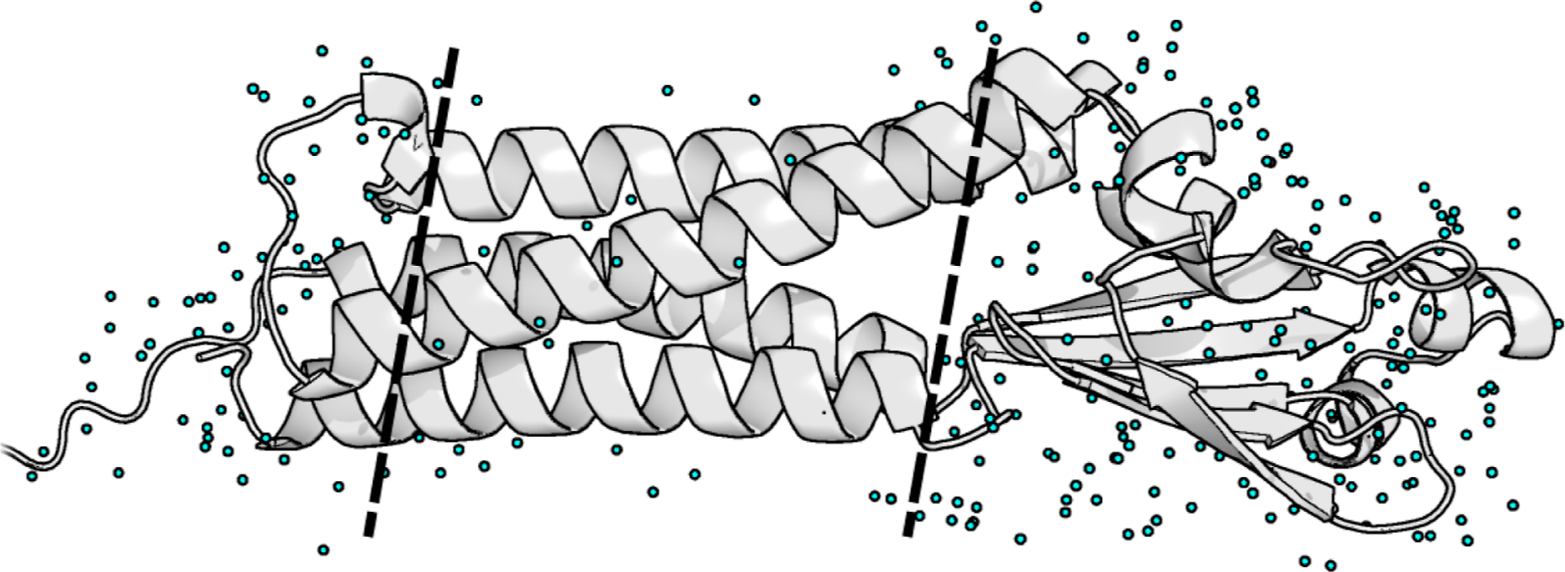
Hydration site predictions for membrane-bound protein Clarin-2 generated via AlphaFold3. The predictions show that they are robust to different molecular environments and can accurately predict the number of waters depending on the environment. In the hydrophobic, membrane-bound core of the protein, there are very few predicted waters. However, the hydrophilic intra- and extra-cellular sides had a large number of predicted sites.

One exciting new opportunity to test our model is on the host of computationally predicted structures that have not been experimentally determined. This is especially interesting since leading protein structure methods, such as AlphaFold3 and RosettaFold-AllAtom, do not consider water molecules in their predictions.^52,53^ Thus, there exist thousands of protein structures for which important water locations cannot be predicted easily. To demonstrate this advantage, we used AlphaFold3 to predict the structure of a membrane-bound protein Clarin-2, which has no structure contained in the PDB. The structure of membrane-bound proteins are often difficult in general to predict due to their inability to crystallize, although recent advances have enabled the determination of some membrane-bound proteins.^54,55^ The Clarin-2 protein is essential for hearing function by maintaining stereocilia integrity and could have therapeutic applications.^56,57^ The AlphaFold3 structure seems reasonable according to the confidence metrics and visual inspection; the structure contains the expected four transmembrane domains as well as interfaces on the intra and extra-cellular sides.^58^ An interesting finding was observed after prediction of the water locations, shown in Figure 8. A clear distinction in the number of predicted waters can be seen between the exposed intra/extra-cellular regions and the membrane-bound region. This result demonstrates the ability of our model to not only accurately predict the locations of waters surrounding a protein but to also accurately predict their likelihood. Furthermore, it shows that the model is able to capture essential aspects of physics such as hydrophobicity.

#### 4.3.5 Major Urinary Protein

The free energy of desolvation is a major driving force for protein–ligand binding. In order to test the performance of our thermodynamic profiling in the context of ligand binding, 12 cocrystallized ligands to MUP were overlaid with the predicted HS and the free energy of desolvation was calculated based on the displaced water molecules (Table 6).

**Table 6:** Desolvation Free Energies from Hydration Site Displacement of 12 MUP Ligands. Free Energies are Reported in kJ/mol^a^^,^^b^

PDB ID	1ZND	1ZNE	1ZNG	1ZNH	1ZNK	1QY1	1QY2	1IO6	1I06	1I06	1IO6	1I06
ligand	PE9	HE2	HE4	OC9	F09	IBMP	IPMP	SBT	PT	IPT	ET	MT
WATsiteNN	–24.4	–27.6	–33.1	–36.2	–34.8	–40.3	–35.3	–35.3	–32.0	–31.0	–29.8	–26.5
WATsite	–23.4	–27.2	–23.2	–36.4	–36.4	–36.8	–41.1	–35.9	–39.4	–35.9	–29.5	–21.6
experimental	–23.1	–28.3	–32.5	–35.6	–38.8	–38.5	–33.9	–35.3	–34.3	–32.6	–29.2	–24.2
MM-GB/SA	–33.9	–39.0	–27.2	–23.2	–38.1	–30.2	–28.2	–38.4	–34.7	–34.2	–30.5	–28.9

a Shows the transformed predictions obtained by a linear regression (of slope one) of the experimental values on the model predictions.

b For ligands following SBT, structures were obtained by removing specified carbon atoms from the crystal structure of SBT. We named our model “WATsiteNN” here.

**Figure 9:**
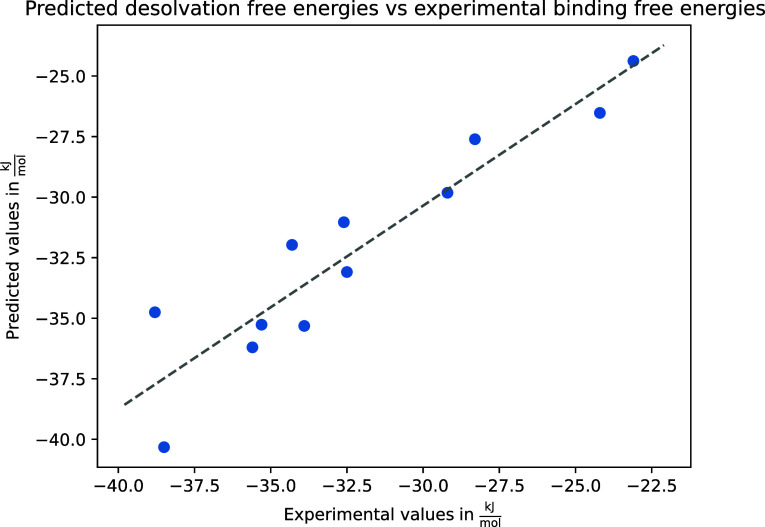
Regression plot comparing predicted desolvation free energy and binding free energy.

While the desolvation free energy is only one component of ligand binding, these ligands are largely hydrophobic and share a single carboxylic acid moiety, and therefore, the predominant difference between them is the number and position of displaced water molecules. Binding free energy estimations using the MM-GB/SA method were obtained from previous studies.^39,59^Figure 9 shows a regression plot between the predicted desolvation energies for all ligands in the data set and the experimentally measured values. At a water displacement tolerance level of 2.4 Å, we obtain an *R*^2^ value of 0.867.

This is significantly superior to MM-GB/SA results we obtained for the same complexes, where almost no correlation was found (see Table 7).

**Table 7:** Coefficient of Determination *R*^2^ and RMSE for Fitting the Experimental Binding Free Energies^a^

	WATsite	WATsiteNN	MM-GB/SA
***R***^2^	0.63	0.87	6.4 × 10^–5^
**RMSE**	4.0	1.8	6.8

a We named our model “WATsiteNN” here.

## 5 Conclusions

We introduced a new method for hydration site prediction and thermodynamic profiling based on an equivariant deep neural network trained on a large corpus of MD data. Our results demonstrate that our model is fast, accurate, and robust to chemical and biological perturbations. Other hydration site profiling tools can take several hours for just a single protein conformation due to the expensive explicit-water simulations. However, by leveraging our model, we can make predictions on the seconds time scale, enabling high-throughput hydration site prediction and profiling. This is a crucial advancement to predict HS for flexible proteins in different conformations or for the integration of hydration site prediction into deep learning models for molecular structure prediction. Our results further demonstrated that the model is highly accurate and can reproduce results from MD and experimentally resolved structures with high fidelity. Furthermore, thermodynamic predictions from our model showed strong agreement with those obtained from MD and experimentally measured affinities for a series of ligands. We investigated the robustness and generalization of our model, since this is a common failure mode seen in deep learning models. Specifically, we evaluated the model under different perturbations to the protein input such as conformational changes and single-point mutations and then confirmed our predictions against the known water locations. The model showed no signs of overfitting and was capable of responding accurately to small changes in the overall protein structure. In summary, our model offers several key advantages over existing hydration site prediction tools. The ability to quickly and accurately predict hydration site locations and thermodynamics has a range of potential applications, from small molecule drug design and lead optimization to peptide and protein design. Our model could also be integrated with other emerging technologies such as deep learning models for cofolding, dynamics, and free energy estimation.

## Data Availability

The code for this work is provided here: https://github.com/lillgroup/HydrationSitePrediction. The data is available here: https://zenodo.org/records/14182834

## Supporting Information Available

Further results on the training and test set, correlation plots on the training set, and definition of evaluation metrics (PDF)
