# Supplementary material for: Accelerated Hydration Site Localization and Thermodynamic Profiling
Source: J Chem Inf Model. 2025 Feb 28;65(6):2794–805. doi: 10.1021/acs.jcim.4c02349 (PMC11938278; doi:10.1021/acs.jcim.4c02349)
Supplement: Supplementary file 1 — ci4c02349_si_001.pdf [file ci4c02349_si_001.pdf]

Supporting Information
Accelerated Hydration Site Localization and Thermodynamic Profiling

Florian B. Hinz,[†,‡] Matthew R. Masters,[†,‡] Julia T. Nguyen,[†] Amr H. Mahmoud,[†] and Markus A. Lill[*,†]

†*Department of Pharmaceutical Sciences, University of Basel, Basel, Switzerland*

‡*These authors contributed equally to this work.*

E-mail: markus.lill@unibas.ch

Graphics

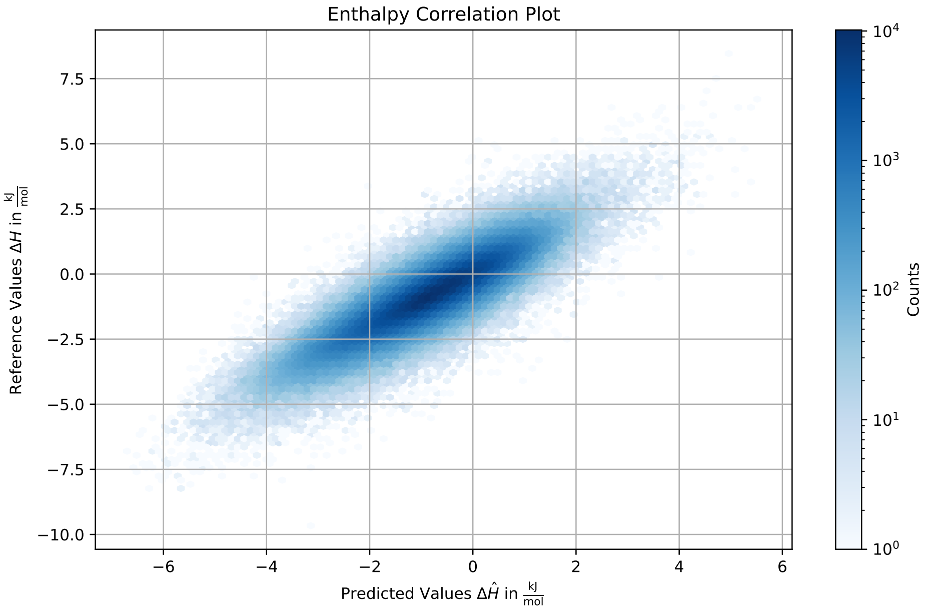

Figure S1: Hexbin plot showing the correlation between the enthalpy predictions and the enthalpy values obtained from WATsite for the train set.

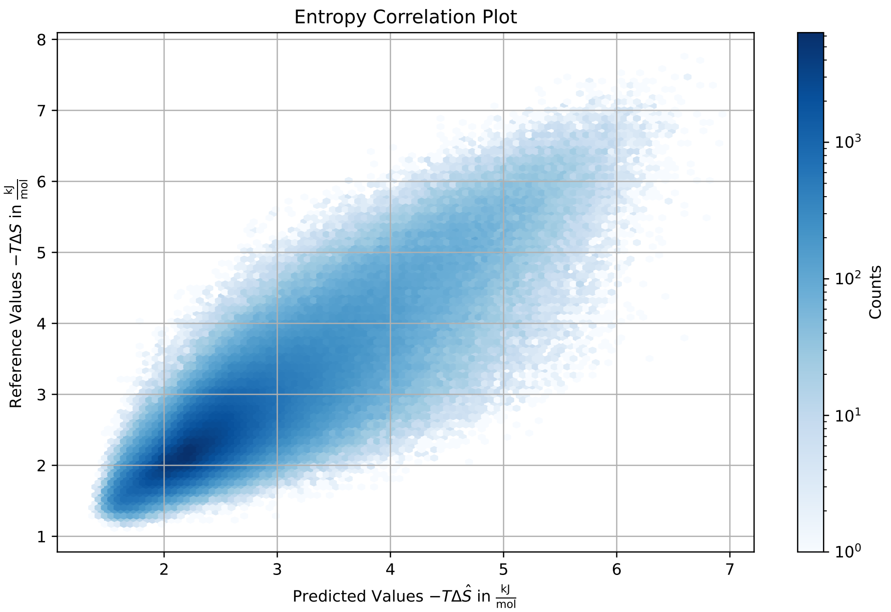

Figure S2: Hexbin plot showing the correlation between the entropy predictions and the entropy values obtained from WATsite at $T = 300$ K for the train set.

Tables

Further results on test set

Table S1: Second layer waters: Ground truth recovery rate at different occupancy levels

	**Occupancy**				
	**[0.5,0.6]**	**[0.6,0.7]**	**[0.7,0.8]**	**[0.8,0.9]**	**[0.9,1.0]**
**Cutoff**					
$r = 0.5$	11.4%	17.6%	22.8%	29.4%	38.5%
$r = 1.0$	20.8%	30.6%	39.8%	50.7%	63.5%
$r = 1.5$	26.6%	38.0%	48.7%	59.4%	74.5%
$r = 2.0$	33.3%	45.6%	56.2%	68.2%	82.2%

Results on train set

For reference we provide the results for the training set as well.

Table S2: Ground truth recovery rate and prediction hit rate

**Cutoff**	**Ground Truth Recovery Rate**	**Prediction Hit Rate**
0.5	62.7%	50.2%
1.0	82.1%	66.0%

Table S3: Ground truth recovery rate (GTRR) for first and second layer

**Cutoff**	**GTRR first layer**	**GTRR second layer**
0.5	66.2%	16.7%
1.0	86.3%	28.2%

Evaluation measures

In order to evaluate the quality of the hydration site coordinate predictions of our model we need to define two evaluation measure: One measuring how many true hydration sites were

Table S4: Ground truth recovery rate at different occupancy levels

	Occupancy				
	**[0.5,0.6]**	**[0.6,0.7]**	**[0.7,0.8]**	**[0.8,0.9]**	**[0.9,1.0]**
**Cutoff**					
$r = 0.5$	45.3%	61.1%	69.2%	74.6%	75.4%
$r = 1.0$	65.1%	81.8%	89.1%	93.0%	93.3%
$r = 1.5$	72.2%	87.5%	93.6%	96.6%	95.9%
$r = 2.0$	76.2%	90.1%	95.4%	97.8%	96.7%

Table S5: First layer waters: Ground truth recovery rate at different occupancy levels

	Occupancy				
	**[0.5,0.6]**	**[0.6,0.7]**	**[0.7,0.8]**	**[0.8,0.9]**	**[0.9,1.0]**
**Cutoff**					
$r = 0.5$	51.7%	64.4%	70.9%	75.3%	75.6%
$r = 1.0$	73.5%	85.8%	90.9%	93.8%	93.4%
$r = 1.5$	80.9%	91.5%	95.3%	97.3%	96.0%
$r = 2.0$	84.5%	93.7%	96.9%	98.3%	96.8%

Table S6: Second layer waters: Ground truth recovery rate at different occupancy levels

	Occupancy				
	**[0.5,0.6]**	**[0.6,0.7]**	**[0.7,0.8]**	**[0.8,0.9]**	**[0.9,1.0]**
**Cutoff**					
$r = 0.5$	12.3%	19.8%	27.8%	35.5%	39.6%
$r = 1.0$	21.6%	32.8%	44.8%	54.5%	64.5%
$r = 1.5$	27.3%	39.1%	52.2%	63.1%	72.8%
$r = 1.5$	33.4%	46.1%	58.9%	69.5%	79.2%

Table S7: Coefficient of determination (R^2) and Means Squared Error (MSE) between prediction and ground truth for both enthalpy and entropy difference on the training set

	$-T\Delta S$	ΔH
**MSE** in $\frac{\text{kJ}^2}{\text{mol}^2}$	0.19	0.28
$\mathbf{R}^2$	0.75	0.76

discovered and a second one setting this into context by measuring how many predictions were reasonable.

Let $m, n \in \mathbb{N}$ and $(a_i)_{i=1}^m \in \mathbb{R}^3$ be the "ground truth" points and $(b_j)_{j=1}^n \in \mathbb{R}^3$ be the "predictions". Let $r \in \mathbb{R}_+$ be the "cutoff radius". Let us agree to the following definitions

$$L^r_{GTRR} := \frac{1}{m}\sum_{i=1}^{m}\left(1-\prod_{j=1}^{n}\mathbb{1}_{]r,\infty[}(||a_i-b_j||_2)\right) \quad (1)$$

$$L^r_{PHR} := \frac{1}{n}\sum_{j=1}^{n}\left(1-\prod_{i=1}^{m}\mathbb{1}_{]r,\infty[}(||a_i-b_j||_2)\right) \quad (2)$$

We refer to L^r_{GRR} as the **G**round **T**ruth **R**ecovery **R**ate (**GTRR**) and L^r_{PHR} as the **P**rediction **H**it **R**ate (**PHR**).

In words, the *ground truth recovery rate* is the proportion of ground truth elements that have at least one prediction within a radius r. The *prediction hit rate* is the proportion of predictions that are within a radius r of *any* ground truth element.
